# Movement of anterior teeth using clear aligners: a three-dimensional, retrospective evaluation

**DOI:** 10.1186/s40510-018-0207-3

**Published:** 2018-04-02

**Authors:** Michele Tepedino, Valeria Paoloni, Paola Cozza, Claudio Chimenti

**Affiliations:** 1 Department of Biotechnological and Applied Clinical Sciences, University of L’Aquila, Viale S.Salvatore, Edificio Delta 6, 67100 L’Aquila, Italy; 2 Department of Clinical Sciences and Translational Medicine, University of Rome Tor Vergata, Viale Oxford 81, 00133 Rome, Italy

**Keywords:** Clear aligners, Orthodontic tooth movement, Torque

## Abstract

**Background:**

Clear aligner treatment offers several advantages, but the available literature shows that some kind of tooth movements are unpredictable. In addition, the majority of the studies are focused on one clear aligner system, while different characteristics of various systems can provide different treatment outcomes. The aim of the present retrospective cohort study was to evaluate the predictability of Nuvola® aligner system in achieving torque movements of anterior teeth.

**Methods:**

Thirty-nine adult patients, who were consecutively treated with clear aligners, were retrospectively selected, and digital models pre-treatment (T0), post-treatment (T1) and the digital setup models (TS) were collected. Only the first phase of treatment made of 12 aligners was considered for the present study. Torque of anterior teeth was measured as labiolingual inclination on digital models at T0, T1, and TS using VAM software. Any difference between the predicted and achieved torque movements was evaluated using Wilcoxon signed-rank test or paired sample *t* test. First-type error was set as *p* < 0.008.

**Results:**

No statistically significant difference was found for all the anterior teeth between predicted and achieved torque movements.

**Conclusions:**

The studied clear aligner system was able to produce clinical outcomes comparable to the planning of the digital setup relative to torque movements of the anterior teeth.

**Electronic supplementary material:**

The online version of this article (10.1186/s40510-018-0207-3) contains supplementary material, which is available to authorized users.

## Background

Since Kesling [1] first proposed the use of sequential thermoformed aligners for orthodontic tooth movement and Align Technology (Santa Clara, CA, USA) in 1997 developed this idea into a feasible treatment modality with the introduction of CAD/CAM technologies [2], clear aligner therapy has experienced increasing diffusion. The success of clear aligner therapy probably relies on some advantages like aesthetics, greater comfort for the patient [3], and improved oral hygiene and periodontal health [4] compared to fixed appliances. Growing demand led to improvement of the technique, which is no longer limited to simple crowding cases but extended also to complex malocclusions [5].

The first study on the efficacy of Invisalign® (Align Technology, Santa Clara, CA, USA) clear aligners reported a mean accuracy—defined as the overlap between the predicted and achieved movements—of 41% overall [6], while subsequent study reported more promising results [7]. However, there is still an open debate about the predictability of clear aligner therapy, especially regarding complex tooth movements. While levelling and aligning, intrusion, and bodily distalisation of upper molars of no more than 1.5 mm were demonstrated to be predictable movements, rotations, extrusion, and torque movements are difficult to achieve with clear aligners [8–11]. In addition, most of the available studies are relative to the Invisalign system, but differences in the aligner’s material properties and thickness, the production process, the model’s precision, and the position of the aligner’s margin all have an effect on the final performance of the appliance [12–15]; therefore, different results can be expected from different clear aligner systems [16]. Recently, Lombardo et al. [16], comparing planned and achieved tipping and rotation tooth movements in patients using another clear aligners system, found that orthodontic aligners are unable to achieve programmed movement with 100% predictability. In particular, although tipping movements were efficaciously achieved, especially at the molars and premolars, rotation of the lower canines was an unpredictable movement.

To the best of our knowledge, there is a lack of reliable literature on this subject, especially on the effective clinical predictability of torque movement of the anterior teeth using different clear aligners. Therefore, the aim of the present study was to evaluate the efficiency of a clear aligner system (Nuvola®, GEO S.r.l., Rome, Italy) [17] in controlling the torque movement of upper and lower anterior teeth. The null hypothesis was that no difference exists between planned and achieved torque movements.

## Methods

The records of patients referred to the Dental Clinic of the Department of Biotechnological and Applied Clinical Sciences, University of L'Aquila, from September 2013 to September 2017 for orthodontic treatment with clear aligners, were screened for the following inclusion criteria:

- Caucasian adult patients (> 18 years) with full permanent dentition;
- Up to 6 mm of crowding in the anterior segment of the arch (from the distal right canine to distal left canine) evaluated on dental casts according to Lundstrom [18]
- Non-extractive orthodontic treatment with Nuvola® aligners;
- Presence of retention attachments for the buccal surfaces of the first and second premolars;
- Treatment plan that required interproximal reduction (IPR);
- Absence of local and systemic conditions that can alter bone metabolism.

Sample size calculation (G*Power version 3.1.9.2, Universitat Dusseldorf, Germany) [19], according to data on torque measurements retrieved from a previous study on the Invisalign® system [7], revealed that having a difference in the response of matched pairs of 0.26 and a standard deviation of the difference of 0.49; fifty-eight pairs would be needed to be able to reject the null hypothesis with a power of 0.90 and a type I error of 0.008.

Thirty-nine patients (14 males, 25 females) with a mean age of 30.7 years were retrospectively enrolled in the study group (Table 1), for a total of 63 treated dental arches (36 upper and 27 lower dental arches). Informed consent was obtained from every patient before inclusion.

**Table 1:** Demographic composition of the study sample

	Males (*n* = 14)	Females (*n* = 25)	Total (*n* = 39)
Age (mean ± SD)	30.7 ± 8.5	30.7 ± 10.8	30.7 ± 9.3

Age is expressed in years

### Orthodontic treatment protocol

All subjects underwent a non-extractive orthodontic treatment with Nuvola® aligners. The Nuvola® system is designed to proceed through consecutive steps of a maximum of 12 aligners. After each step, new impressions should be acquired to design a new setup and to move forward to the next treatment phase.

The treatment plan required the presence of retention attachments for the buccal surfaces of the first and second premolars and the IPR of anterior teeth in order to achieve the correct dental alignment. Patients were instructed to wear their aligners for 22 h per day, except during meal times and oral hygiene procedures. Aligners were replaced every 14 days.

### Measurement of digital models

For each treated dental arch, pre-treatment (T0), real post-treatment (T1: at the end of the first phase of treatment after 12 aligners), and ideal post-treatment according to setup (TS) digital casts were available. Pre-treatment and post-treatment models were acquired using a 3Shape E1 scanner (3Shape, Copenhagen, Denmark), and setups were constructed using Maestro 3D Ortho Studio software (AGE Solutions S.r.l., Pisa, Italy). The digital models of upper and lower arches at T0, T1, and TS were acquired as .STL files [20] and analysed by a single operator (M.T.) using VAM software (Vectra, Canfield Scientific, Fairfield, NJ, USA).

**Fig. 1:**
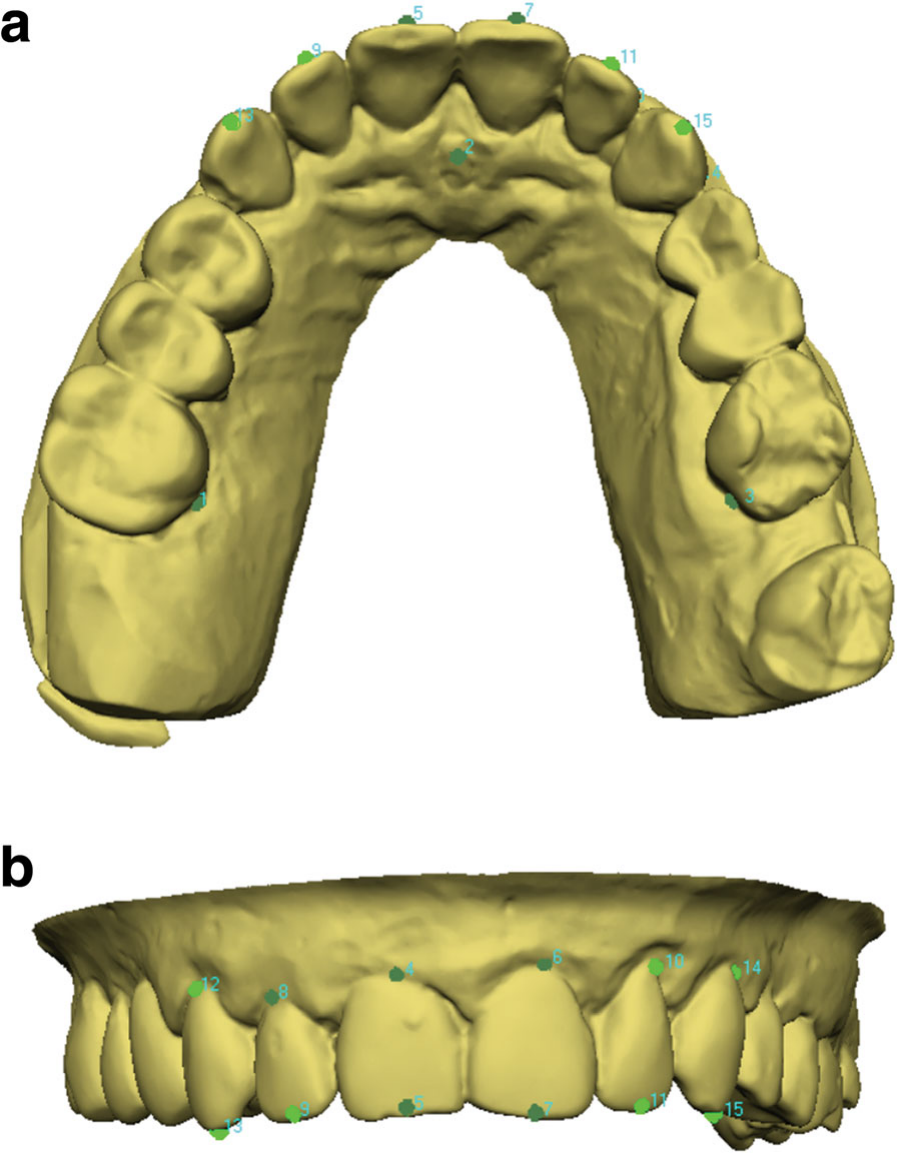
Torque measurement using VAM software. Reference landmarks and points defining the labial FACC of the anterior teeth were localised on every digital model. **a** Occlusal view and **b** frontal view

Fifteen landmarks were localised by the same well-trained operator on each digital model: the gingival limit of the lingual facial axis of clinical crown (FACC) of the right and left first molars, the incisal spot/the papilla between the central incisors (for upper and lower arch, respectively), and the gingival and occlusal limit of the buccal FACC of the central incisors, lateral incisors, and canines (Fig. 1). The landmarks’ coordinates were exported as a .txt file and then imported into an Excel spreadsheet (Microsoft Excel, Microsoft, Redmond, WA, USA). The gingival points of the right and left molars and the point on the incisal spot/the papilla between the central incisors were used to define a reference plane. Then, the coordinates of the gingival and occlusal points of the buccal FACC of each tooth were transformed according to this reference plane. Torque was measured as the labiolingual inclination of the FACCs relative to the reference plane [21]. The torque angles were then calculated using trigonometry.

### Error of the method

Thirty dental arches were randomly selected using online software (https://www.randomizer.org/), and landmark selection and torque measurements were repeated by the same operator after 2 weeks. For all measurements, Dahlberg’s formula (*s* =  √ (*Sd* ^ 2 )/2*n*, where *d* = difference between the first and second measurements) was used to calculate the standard error on the repeated sets of measurements. Bland–Altman plots were used to check for the intra-observer reliability between the two sets of measurements [22].

### Statistical analysis

A Shapiro–Wilk normality test was used to analyse the type of data distribution for all the variables. A paired sample *t* test or a Wilcoxon signed-rank test was used to evaluate if any statistically significant difference was present between the predicted movements (TS) and the clinically achieved torque (T1). First-type error was set as 0.008 after applying Bonferroni correction for multiple testing. Descriptive statistics including mean and standard deviation were also computed for all the variables.

## Results

**Fig. 2:**
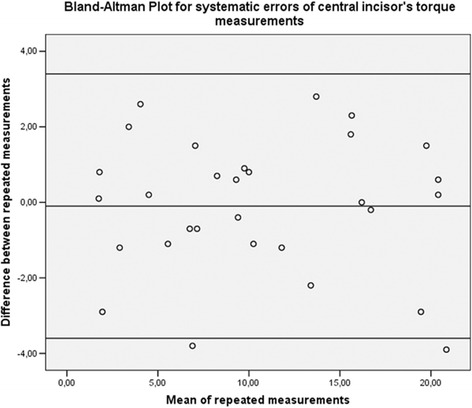
Bland–Altman plot for repeated measurements of central incisors, confirming the absence of systematic errors

**Fig. 3:**
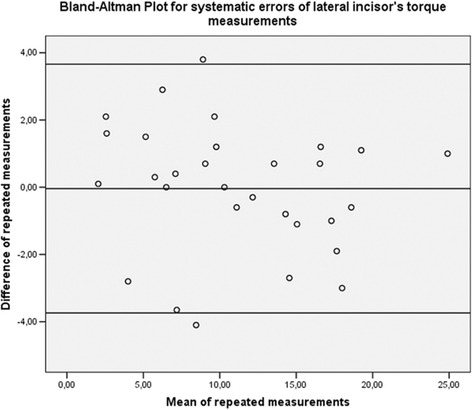
Bland–Altman plot for repeated measurements of lateral incisors, confirming the absence of systematic errors

**Fig. 4:**
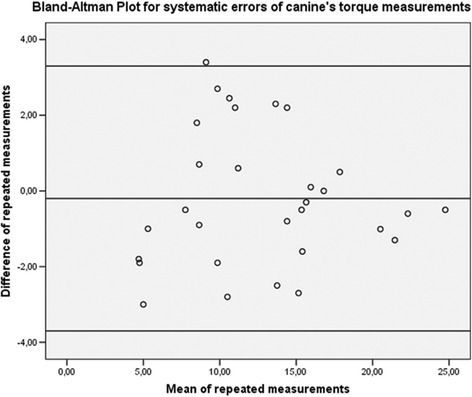
Bland–Altman plot for repeated measurements of canines, confirming the absence of systematic errors

Regarding the error of the method, the standard error was 1.2° for central incisor’s torque, 1.3° for lateral incisor’s torque, and 1.3° for canine’s torque. Bland–Altman plots revealed no systematic errors, confirming the intra-observer reliability of the measurements (Figs. 2, 3, and 4).

Descriptive statistics are reported in Table 2. The mean predicted torque movement (predicted = TS–T0) was 2.3° ± 2.5 for left central incisor, 2.3° ± 2.4 for left lateral incisor, 2.6° ± 2.6 for left canine, 2.4° ± 2.4 for right central incisor, 3.1° ± 2.9 for right lateral incisor, and 2.8° ± 2.8 for right canine in the upper arch; in the lower arch, the mean predicted torque movement was 2.3° ± 1.7 for left central incisor, 3.1° ± 2.7 for left lateral incisor, 3.2° ± 3.1 for left canine, 1.7° ± 1.7 for right central incisor, 3.2° ± 3.6 for right lateral incisor, and 2.2° ± 4.0 for right canine. Increasing angular values means that the tooth’s crown was moved labially, while decreasing values were measured when the crown moved lingually.

**Table 2:** Descriptive statistics for torque measurements

			T0	T1	TS
Upper arch (*n* = 36)	Left side	Canine	12.7 ± 5.7	11.4 ± 4.5	11.6 ± 4.9
		Lateral incisor	10.7 ± 5.6	8.8 ± 4.9	9.4 ± 5.5
		Central incisor	9.4 ± 5.4	7.7 ± 4.5	9.1 ± 5.8
	Right side	Canine	12.0 ± 4.9	10.6 ± 5.2	9.9 ± 4.5
		Lateral incisor	9.3 ± 4.8	8.1 ± 4.4	8.5 ± 4.8
		Central incisor	8.9 ± 5.7	7.7 ± 4.3	8.3 ± 5.5
Lower arch (*n* = 27)	Left side	Canine	14.3 ± 6.2	11.8 ± 4.9	11.6 ± 5.4
		Lateral incisor	11.0 ± 4.8	8.6 ± 3.8	8.6 ± 4.3
		Central incisor	11.0 ± 4.9	9.3 ± 5.1	9.9 ± 4.6
	Right side	Canine	16.2 ± 6.1	14.3 ± 5.8	15.0 ± 5.7
		Lateral incisor	8.1 ± 4.7	7.8 ± 3.9	7.5 ± 3.7
		Central incisor	9.8 ± 4.5	9.4 ± 4.8	9.1 ± 4.2

Torque is expressed in degrees (°)

The paired sample *t* test and Wilcoxon signed-rank test revealed that no statistically significant difference was present between the torque measurements at TS and T1; therefore, the null hypothesis was accepted, confirming that the predicted movements were generally achieved (Table 3). The dataset containing all the collected measurements is attached as Additional file 1.

**Table 3:** Paired *t* test for the comparison of the predicted (TS) and achieved (T1) torque

			Mean difference	Standard error	*p*	95% confidence interval	
						Lower bound	Upper bound
Upper arch (*n* = 36)	Left side	Canine	0.21*	0.52	0.697	− 1.26	0.85
		Lateral incisor	− 1.57^†^	–	0.116	–	–
		Central incisor	− 1.86^†^	–	0.063	–	–
	Right side	Canine	− 1.0^†^	–	0.318	–	–
		Lateral incisor	− 0.44*	0.5	0.437	− 1.59	0.70
		Central incisor	− 0.73^†^	–	0.466	–	–
Lower arch (*n* = 27)	Left side	Canine	0.21*	0.67	0.753	− 1.16	1.59
		Lateral incisor	0.03*	0.72	0.965	− 1.46	1.52
		Central incisor	− 0.71*	0.66	0.289	− 2.07	0.64
	Right side	Canine	− 0.67*	0.91	0.472	− 2.54	1.21
		Lateral incisor	0.38*	0.91	0.683	− 1.50	2.25
		Central incisor	0.25*	0.55	0.654	− 0.89	1.39

Torque values are expressed as degrees (°)

*Paired sample *t* test

†Z statistics from Wilcoxon signed-rank test

## Discussion

The aim of the present study was to evaluate the predictability of the Nuvola® aligner system in performing torque movements on the anterior teeth, and the results showed that the movements predicted from the digital setup were generally achieved.

It was decided to include only adult patients in the study group because they represent most of the patients who require orthodontic treatment with invisible techniques, and because those patients generally show a better compliance, compared to adolescents [3, 23], thus reducing a possible source of bias. In addition, only patients with mild crowding were included because clear aligner treatment is not yet recommended for complex malocclusions [8].

Regarding overall treatment efficiency, some studies evaluated the outcomes of clear aligner treatment versus fixed appliance treatment using the discrepancy index of the American Board of Orthodontics or the Peer Assessment Rating (PAR) index [24]. Some authors showed that Invisalign® produced significantly lower scores than fixed appliances and was unable to correct buccolingual inclination, occlusal contacts, occlusal relationships, and overjet [25], while other authors concluded that there were no statistically significant differences in the achieved results [26]. The different conclusions of these two cited articles, which were published 12 years apart, probably reflect the development and improvements in materials, technologies, and treatment protocols.

Regarding the efficiency of the single type of tooth movements, a systematic review of the literature demonstrated that rotations, especially of teeth with a rounded shape like canines and premolars; extrusions; and bodily translations are less predictably achieved with clear aligners [8]. On the other hand, it was proved that intrusions and molar distalisations of up to 1.5 mm can be efficiently achieved [8].

Few articles are available in the literature regarding torque movements with clear aligners [7, 10, 11, 27, 28]. Torque movement requires the presence of a force couple acting on the tooth, and the biomechanics of this type of tooth movement was described by Hahn et al. [10]: the movement inside each aligner determines that initially the aligner does not fit the tooth crown perfectly, and a certain amount of reversible deformation is present at the gingival margins of the aligner. Such deformation prevents the formation of an effective couple [10] and is the reason why torque is difficult to achieve with clear aligners. This was confirmed by Elkholy et al. [29] measuring the moment/force (M/F) ratio produced during the bodily movement of an upper central incisor with a different aligner thickness and amount of movement for each aligner; the authors found that the M/F ratio was not large enough to produce an effective counter-movement in any of the situations studied. Zhang et al. [28] combined information from digital models and cone-beam CT images to evaluate both crown and root position during anterior teeth movements. Interestingly, the authors found a large amount of crown movement and a small amount of root movement, suggesting that clear aligners are capable of mainly crown tilting movements.

When evaluating the outcomes of clear aligner treatment, it should be considered that several factors play a role in determining successful tooth movement: the attachment’s shape and position [12], the aligner’s material and thickness [14, 30], the amount of activation present in each aligner [31], and the techniques used for the production of the aligners [15]. To be thorough, treatment outcomes depend also on the patient’s characteristics, bone density and morphology [32], and crown and root morphology of the teeth [33], as well as on factors related to the clinician, like the accuracy in performing the requested amount of IPR, which is usually underestimated [34].

With these considerations in mind, it can be argued that not all clear aligner systems could provide the same efficiency and predictability. This is of great importance because many systems are available on the market, but the majority of the available studies are focused on Invisalign® aligners. Therefore, the generalisability of the results of those studies can be questioned.

The results of the present study showed that with Nuvola® aligners, the torque movements for central and lateral incisors and canines of both arches predicted in the digital setup were, in general, clinically achieved; the mean difference between predicted (TS) and achieved (T1) torque was between 0.03° and 1.86°and was not statistically significant (Table 3). This finding is in contrast to the results presented by Simon et al. [27], who found a significant difference between the planned and achieved torque for an upper incisor with Invisalign® for a movement greater than 10°, measuring a movement accuracy of 51.5% using Power Ridges and of 41.9% using only ellipsoid attachments. On the other hand, Lombardo et al. [16] found results comparable to those of the present study with another clear aligner system, showing a mean accuracy of 72.9% and no statistically significant difference between planned and achieved torque for some teeth, but not for all. It must be underlined that the results of the present study refer to the end of a phase of 12 aligners, and not to the treatment’s end. This is because the studied system proposes a clear aligner treatment that progresses through subsequent steps, each one comprising a maximum of 12 aligners; at the end of each phase, new impressions are taken, and a new digital setup is prepared. The maximum torque movement planned was 12.7° in the upper arch (mean 2.6°) and 20.8° in the lower arch (mean 2.7°) for cases presenting mild to moderate crowding, and those amounts of torque were easily achieved clinically.

All the available studies agree that there is always a certain amount of discrepancy between the digital setup and the real clinical outcome: the deformation of the aligner when accommodated in its position produces areas of contact with the teeth and gaps where the aligner is not touching the teeth surfaces, and this reduces the efficacy in producing tooth movements [33]. As the treatment progresses and aligners are changed, the fit between the aligner and the teeth becomes progressively looser because tooth movements are not properly achieved. On the other hand, the present study demonstrated that small amounts of movement for a short series of aligners can be predictably achieved.

Torque measurements were performed following a protocol that was validated previously [21]: the use of a constructed reference plane that can be only minimally altered during orthodontic treatment allows for precise measurements. The authors who proposed this method reported that the average method errors of the torque values for all teeth were 1.2° and 1.5° for the mandible and the maxilla, respectively [21]. In the present study, the measured error of the method was between 1.2° and 1.3°, corresponding to the lower bound of the range reported by Huanca Ghislanzoni et al. [21].

The main limitation of the present study is its retrospective design; however, care was taken to minimise any selection bias, since all the included subjects were treated consecutively during the considered timespan and were chosen only according to the predetermined inclusion criteria. It was not possible to blind the operator who made the measurements given the nature of the study because it would have been easy to recognise and understand the sequence of the digital models. The results of the present study suggest that an aligner system that prescribes subsequent steps of treatment, with a re-evaluation of the case and a new digital setup at each step, can offer a clinical advantage in terms of predictability of tooth movement, especially considering that the new setups do not have additional costs for the clinician, except for the impression material and the chairside time needed to make new impressions. Further studies are needed to evaluate if a step-by-step progression is effective in producing a final treatment outcome that better corresponds to the digital setup with respect to other aligner systems.

## Conclusions

For patients with moderate crowding up to 6 mm, the Nuvola® clear aligner system was able to produce clinical outcomes comparable to the planning of the digital setup relative to torque movements of the anterior teeth. Considering subsequent treatment steps of 12 aligners each, this allowed achievement of predictable torque movements.

## Additional file

Additional file 1: The dataset containing all the collected measurements. (XLSX 27 kb)

## Abbreviations

FACC: Facial axis of clinical crown
RME: Rapid maxillary expansion

## References

[1] Kesling HD (1946). Coordinating the predetermined pattern and tooth positioner with conventional treatment. Am J Orthod Oral Surg.

[2] Kuo E, Miller RJ (2003). Automated custom-manufacturing technology in orthodontics. Am J Orthod Dentofac Orthop.

[3] Lin F, Yao L, Bhikoo C, Guo J (2016). Impact of fixed orthodontic appliance or clear-aligner on daily performance, in adult patients with moderate need for treatment. Patient Prefer Adherence.

[4] Rossini G, Parrini S, Castroflorio T, Deregibus A, Debernardi CL (2015). Periodontal health during clear aligners treatment: a systematic review. Eur J Orthod.

[5] Baldwin DK, King G, Ramsay DS, Huang G, Bollen A (2008). Activation time and material stiffness of sequential removable orthodontic appliances. Part 3: premolar extraction patients. Am J Orthod Dentofac Orthop.

[6] Kravitz ND, Kusnoto B, BeGole E, Obrez A, Agran B (2009). How well does Invisalign work? A prospective clinical study evaluating the efficacy of tooth movement with Invisalign. Am J Orthod Dentofac Orthop.

[7] Grünheid T, Loh C, Larson BE (2017). How accurate is Invisalign in nonextraction cases? Are predicted tooth positions achieved?. Angle Orthod..

[8] Rossini G, Parrini S, Castroflorio T, Deregibus A (2015). Efficacy of clear aligners in controlling orthodontic tooth movement: a systematic review. Angle Orthod.

[9] Lagravère MO, Flores-Mir C (2005). The treatment effects of Invisalign orthodontic aligners: a systematic review. J Am Dent Assoc.

[10] Hahn W, Zapf A, Dathe H, Fialka-Fricke J, Fricke-Zech S, Gruber R (2010). Torquing an upper central incisor with aligners—acting forces and biomechanical principles. Eur J Orthod.

[11] Simon M, Keilig L, Schwarze J, Jung BA, Bourauel C (2014). Forces and moments generated by removable thermoplastic aligners: incisor torque, premolar derotation, and molar distalization. Am J Orthod Dentofac Orthop.

[12] Dasy H, Dasy A, Asatrian G, Ròzsa N, Lee H-F, Kwak JH (2015). Effects of variable attachment shapes and aligner material on aligner retention. Angle Orthod..

[13] Hahn W, Dathe H, Fialka-Fricke J, Fricke-Zech S, Zapf A, Kubein-Meesenburg D (2009). Influence of thermoplastic appliance thickness on the magnitude of force delivered to a maxillary central incisor during tipping. Am J Orthod Dentofac Orthop.

[14] Lombardo L, Martines E, Mazzanti V, Arreghini A, Mollica F (2012). Stress relaxation properties of four orthodontic aligner materials: a 24-hour in vitro study. Angle Orthod..

[15] Martorelli M, Gerbino S, Giudice M, Ausiello P (2013). A comparison between customized clear and removable orthodontic appliances manufactured using RP and CNC techniques. Dent Mater.

[16] Lombardo L, Arreghini A, Ramina F, Huanca Ghislanzoni LT, Siciliani G (2017). Predictability of orthodontic movement with orthodontic aligners: a retrospective study. Prog Orthod.

[17] Ercoli F, Tepedino M, Parziale V, Luzi C (2014). A comparative study of two different clear aligner systems. Prog Orthod.

[18] Lundstrom A (2007). An investigation of 202 pairs of twins regarding fundamental factors in the aetiology of malocclusion. Eur J Orthod.

[19] Faul F, Erdfelder E, Lang A-G, Buchner A (2007). G*Power: a flexible statistical power analysis program for the social, behavioral, and biomedical sciences. Behav Res Methods.

[20] Paoloni V, Lione R, Farisco F, Halazonetis DJ, Franchi L, Cozza P (2017). Morphometric covariation between palatal shape and skeletal pattern in class II growing subjects. Eur J Orthod.

[21] Huanca Ghislanzoni LT, Lineberger M, Cevidanes LHS, Mapelli A, Sforza C, McNamara JA (2013). Evaluation of tip and torque on virtual study models: a validation study. Prog Orthod.

[22] Bland JM, Altman DG (1986). Statistical methods for assessing agreement between two methods of clinical measurement. Lancet.

[23] Alexandropoulos A, Al Jabbari YS, Zinelis S, Eliades T (2015). Chemical and mechanical characteristics of contemporary thermoplastic orthodontic materials. Aust Orthod J.

[24] Richmond S, Shaw WC, O’Brien KD, Buchanan IB, Jones R, Stephens CD (1992). The development of the PAR index (peer assessment rating): reliability and validity. Eur J Orthod.

[25] Djeu G, Shelton C, Maganzini A (2005). Outcome assessment of Invisalign and traditional orthodontic treatment compared with the American Board of Orthodontics objective grading system. Am J Orthod Dentofac Orthop.

[26] Gu J, Tang JS, Skulski B, Fields HW, Beck FM, Firestone AR (2017). Evaluation of Invisalign treatment effectiveness and efficiency compared with conventional fixed appliances using the Peer Assessment Rating index. Am J Orthod Dentofac Orthop.

[27] Simon M, Keilig L, Schwarze J, Jung BA, Bourauel C (2014). Treatment outcome and efficacy of an aligner technique—regarding incisor torque, premolar derotation and molar distalization. BMC Oral Health.

[28] Zhang XJ, He L, Guo HM, Tian J, Bai YX, Li S (2015). Integrated three-dimensional digital assessment of accuracy of anterior tooth movement using clear aligners. Korean J Orthod.

[29] Elkholy F, Panchaphongsaphak T, Kilic F, Schmidt F, Lapatki BG (2015). Forces and moments delivered by PET-G aligners to an upper central incisor for labial and palatal translation. J Orofac Orthop.

[30] Hahn W, Fialka-Fricke J, Dathe H, Fricke-Zech S, Zapf A, Gruber R (2009). Initial forces generated by three types of thermoplastic appliances on an upper central incisor during tipping. Eur J Orthod.

[31] Kwon JS, Lee YK, Lim BS, Lim YK (2008). Force delivery properties of thermoplastic orthodontic materials. Am J Orthod Dentofac Orthop.

[32] Chisari JR, McGorray SP, Nair M, Wheeler TT (2014). Variables affecting orthodontic tooth movement with clear aligners. Am J Orthod Dentofac Orthop.

[33] Hahn W, Engelke B, Jung K, Dathe H, Fialka-Fricke J, Kubein-Meesenburg D (2010). Initial forces and moments delivered by removable thermoplastic appliances during rotation of an upper central incisor. Angle Orthod..

[34] Johner AM, Pandis N, Dudic A, Kiliaridis S (2013). Quantitative comparison of 3 enamel-stripping devices in vitro: how precisely can we strip teeth?. Am J Orthod Dentofac Orthop.

